# Microbial Risks Caused by Livestock Excrement: Current Research Status and Prospects

**DOI:** 10.3390/microorganisms11081897

**Published:** 2023-07-27

**Authors:** Rashidin Abdugheni, Li Li, Zhen-Ni Yang, Yin Huang, Bao-Zhu Fang, Vyacheslav Shurigin, Osama Abdalla Abdelshafy Mohamad, Yong-Hong Liu, Wen-Jun Li

**Affiliations:** 1 State Key Laboratory of Desert and Oasis Ecology, Xinjiang Institute of Ecology and Geography, Chinese Academy of Sciences, Ürümqi 830011, China; 2 University of Chinese Academy of Sciences, Beijing 100049, China; 3 State Key Laboratory of Biocontrol, Guangdong Provincial Key Laboratory of Plant Resources, School of Life Sciences, Sun Yat-Sen University, Guangzhou 510275, China; 4 Institute of Microbiology, Chinese Academy of Sciences, Beijing 100101, China

**Keywords:** livestock excrement, zoonotic pathogens, ARGs, virulence genes

## Abstract

Livestock excrement is a major pollutant yielded from husbandry and it has been constantly imported into various related environments. Livestock excrement comprises a variety of microorganisms including certain units with health risks and these microorganisms are transferred synchronically during the management and utilization processes of livestock excrement. The livestock excrement microbiome is extensively affecting the microbiome of humans and the relevant environments and it could be altered by related environmental factors as well. The zoonotic microorganisms, extremely zoonotic pathogens, and antibiotic-resistant microorganisms are posing threats to human health and environmental safety. In this review, we highlight the main feature of the microbiome of livestock excrement and elucidate the composition and structure of the repertoire of microbes, how these microbes transfer from different spots, and they then affect the microbiomes of related habitants as a whole. Overall, the environmental problems caused by the microbiome of livestock excrement and the potential risks it may cause are summarized from the microbial perspective and the strategies for prediction, prevention, and management are discussed so as to provide a reference for further studies regarding potential microbial risks of livestock excrement microbes.

## 1. Introduction

Livestock excrement is a major source of environmental pollution and poses significant risks to the environment and public health. As the livestock industries have been increasing rapidly [1], unsurprisingly, the amount of livestock excrement has been increasing accordingly. Reports showed that the annual production of livestock excrement in Finland, the EU-27, China, the UK, Indonesia, the USA, and Iran reached 1.60 × 10^10^ (wet weight, in 2011) [2], 1.50 × 10^9^ tons (dry weight, in 2014) [3], 6.90 × 10^6^ tons (dry weight, in 2015) [4], 8.34 × 10^10^ tons (dry weight, in 2016) [5], 7.00 × 10^10^ tons (dry weight, in 2016) [6], 3.50 × 10^10^ tons (dry weight, in 2016) [2], and 2.00 × 10^9^ tons (dry weight, in 2017) [7], respectively. The enormous amounts of livestock excrement cause various environmental and health problems due to the diverse pollutants it contains.

Livestock excrement includes non-biological and biological pollutants. Non-biological pollutants may cause environmental problems including odor emission and a contribution to the greenhouse effect [8], fine particles or particulate matter with an aerodynamic diameter less than 2.5 μm (PM 2.5) [9], enrichment of nitrogen (N), phosphorus (P), potassium (K) in related water sites [10], transmission and enrichment of heavy metals, and pesticides in related soil and water [11]. Due to the extensive expansion of livestock production, these environmental problems caused by livestock excrement pollutants have been increasing dramatically [12,13].

Biological pollutants in livestock excrement mainly pertain to the microorganisms and insects residing in the livestock excrement and their pathogenic/toxic products [14,15]. There are generally trillions of diverse microorganisms residing in the animal intestinal and oral tract [16,17]; these microorganisms are introduced into the livestock excrement and will further enter the relevant environment along with husbandry-related activities [18]. Undoubtedly, the health and environmental problems caused by these issues, in turn, cause significant economic losses, hence, special attention needs to be paid to the risks caused by livestock excrement.

Reports showed that cattle and poultry manures include a wide range of pathogenic microorganisms and parasite eggs which may contribute to the spread of human and animal infectious illnesses [19,20]. Pathogenic strains of certain members of *E. coli*, *Salmonella*, *Bacillus anthracis*, *Shigella*, and *Clostridium botulinum* are frequently found in cattle and poultry manures and may directly or indirectly endanger human health [21]. Those pathogens residing in livestock excrement generally cause health risks due to their spread by direct contact, droplet infection, or consumption of food and water contaminated by livestock excrement-related pathogens [22]. 

Livestock excrement has been used as a fertilizer and even an agent to produce biofuel. Owing to the related human activities, the microbiome of the livestock excrement has been affected and changed accordingly and has also been affecting human health as well. In addition, the distribution of pathogens, such as *Bacillus anthracis* [23], *Francisella tularensis* [24], *Yersinia pestis* [25], *Coxiella burnetii* [26], and *Burkholderia pseudomallei* [27], may cause various infectious diseases in livestock excrement-related samples raised concerns globally [28,29,30]. Therefore, it is urgent to characterize the main features, driving forces, and fate of the the microbiome of livestock excrement so as to predict and prevent any related risks that may occur in the long run.

For the past decades, due to the rapid development of bioinformatics tools, we have gained the general composition and function of animal microbiomes. Nevertheless, there are a limited number of comprehensive reports on the microbial risks that may be caused by livestock excrement. In this review, we highlight the main feature of the microbiome of livestock excrement and elucidate the microbial repertoire and what risks it may cause in humans, animals, and the environment. In summary, this review provides a microbial perspective on the environmental issues arising from the microbiome of livestock excrement and highlights the potential risks associated with it. Furthermore, it explores the prospects of predicting and managing these risks, thereby offering valuable insights for future research in the field of livestock excrement microbial hazards.

## 2. Features of the Microbiome of Livestock Excrement and Microbiome Cycling

The microorganisms observed in livestock excrements include viruses, prokaryotes, and eukaryotes. Being the majority in terms of the total number of species and microorganisms, prokaryotic microorganisms including bacteria and archaea accounting for over 95% of the residing microorganisms, among which archaea comprise only 0.3% to 3% [31,32], while eukaryotes include protozoa and fungi only account for less than 5% [33].

**Figure 1:**
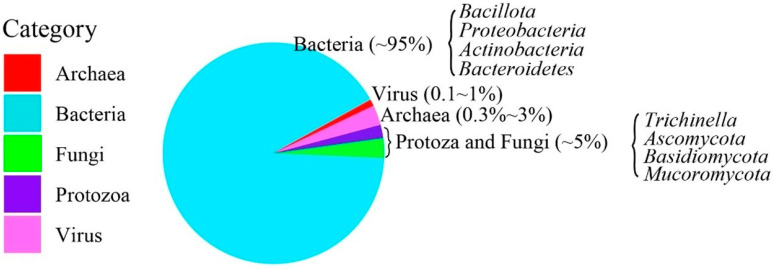
The major representative microbial components of the livestock excrement [33,34,35,36,37,38].

It is well-documented that the major prokaryotic phyla in livestock excrement generally include *Bacillota*, *Bacteroidetes*, *Proteobacteria*, and *Actinobacteria* [34,35,36], while the major fungi phyla include *Ascomycota*, *Basidiomycota*, and *Mucoromycota* [35,37,38]. And there also certain protozoa that reside in livestock manure, including *Cryptosporidia* and *Giardia* [39,40,41], that cause infections in various hosts including humans. The proportion of the major microbial components of livestock excrement are presented in Figure 1. These microorganisms will sustain, transfer, and alter during the related processes of livestock excrement management; it could be regarded as a microbial-cycling process.

**Figure 2:**
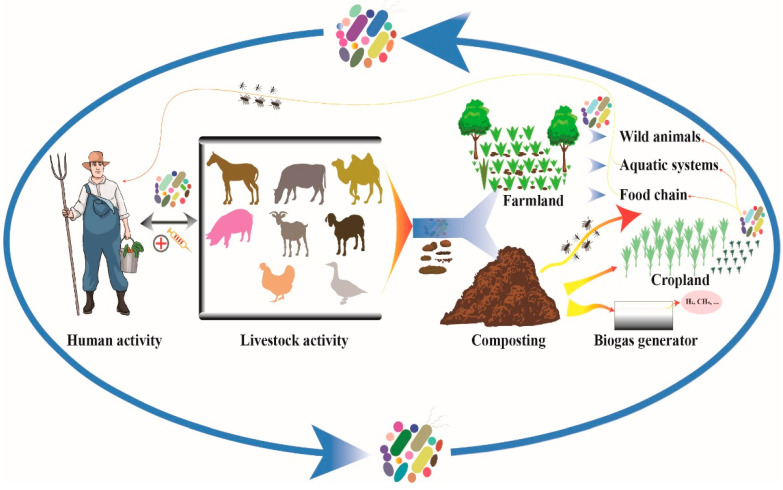
The key processes of livestock microorganisms and included factors. Major factors involved in the livestock excrement microbiome cycling included: (1) contributors of microbiome input: humans, livestock, both domestic and wild animals and insects, and other media of microbes such as feedstock and (2) pathways of microbiome output: composting, livestock activity, airflow, and the activity of humans, animals, and insects.

The cycling process of livestock microorganisms includes the input, output, and forward and reverse transfer of microorganisms across related environments (they will be detailed in the following sections). Moreover, the influencing factors and drivers of the corresponding microbial cycling include human and animal activity and the surrounding environment, which is a complex process with multiple factors. The key processes and factors included are remarked as below (Figure 2).

### 2.1. Input of the Livestock Excrement Microbiome

The input of the livestock excrement microbiome mainly includes the microbiome intruded by the host intestinal microbiome, the microbes from the hosts, and the related both living and non-living environmental components. 

The major contents of the livestock microbiome are from the animal intestinal microbiome [35]. In addition, human activity, such as general farm management, medical treatment of farm animals, and the inclusion of human-related food residuals and food waste as animal feed, also introduces human-related microbiomes into the livestock excrement microbiome [42,43]. Food, water, and insects related to the livestock environment are the second major contributors to the microbiome in livestock excrement [44]. A variety of microorganisms are present in animal feed, including some anaerobic prokaryotic and fungal taxa. On the one hand, along the food chain, these microorganisms are ingested by domestic animals and reside in the intestinal tract and later enter the livestock excrement [45]. On the other hand, feed residues directly become part of livestock excrement. It is noteworthy that the activities of insects associated with domestic animals (such as mosquitoes, flies, fleas, etc.), wild animals (such as mice, *Marmota bobak*, etc.), and birds (such as sparrows, etc.) that live with domestic animals also continuously import microorganisms from the environment and other sources into the livestock living environment, eventually allowing these microorganisms to enter the livestock excrement [46,47,48,49]. In addition, free-range livestock may introduce microorganisms carried by wild animals, especially potential pathogens (such as zoonotic viruses, bacteria, fungi, and parasites) [50,51,52], into livestock niches and in the long run introduce these microorganisms into the livestock excrement, resulting in the movement and enrichment of microorganisms with certain health risks into the closely related environment of domestic animals and humans, which eventually pose a threat to animal and human health.

### 2.2. Output of Livestock Excrement Microbiome

The output pathways of microorganisms in livestock excrement are diverse. Microorganisms use excrement as the medium to survive and transmit, hence their fate is majorly according to the movement of the excrement. For example, the excrement produced by free-range livestock enters the natural environment directly so the relevant microorganisms are also transferred to the natural environment [53,54]. In addition, the excrement produced by livestock in semi-free-range and captivity models are often used as organic fertilizer directly for agricultural production or composted and then used as an organic fertilizer [55]. In recent years, livestock excrement has also been used as a base for biogas digesters and fermentation to produce biogas, such as hydrogen and methane [56,57,58,59]. The composition and structure of microorganisms in treated livestock excrement change during the treatment process. In composting and biogas production, some functional microbial taxa are enriched while the abundance of others decreases. In these processes, some microorganisms are introduced to the surrounding environment by air, related instruments, etc., as the transmission media [60,61]. There are also some spore-producing bacteria and fungi that remain and exist in the processing sites for a long time and may enter the surrounding environment with human activities [62,63,64]. Therefore, if livestock waste is not properly treated and managed, it is likely to cause the rapid spread and enrichment of microorganisms, extremely these disease-causing microorganisms.

### 2.3. Factors Affecting Livestock Excrement Microbiome

Many factors affect the microbial composition of livestock excrement, the main factors of which are the physiology of host animals, the type of feed, human activities, medical interventions, and environmental factors such as the climate [65,66,67]. Different animals have different microbiomes of different taxonomic richness and abundances of each taxon and food plays an important role as the main driver of the animal gut microbe composition in addition to host physiology. Human activities and medical interventions, such as antibiotics, insecticides, fungicides, and other drug treatments, also cause changes in the livestock microbiome and also promote changes in the microbiome of livestock excrement [68,69,70]. The activities of livestock-related wildlife can bring microorganisms from other environments into the microbial niche of livestock waste. Therefore, the need to control and manage the livestock microbiome requires the consideration of all relevant factors. Chemical agents also affect the microbiome composition of livestock excrement, including veterinary drugs in livestock and poultry excrement [71,72]. Antibiotics, pesticides, insecticides, and fungicides are often used to control animal diseases by killing pathogens including parasites, mosquitoes, and flies. Many of these chemical agents remain to be completely metabolized and in the long run will alert the composition of the livestock excrement microbiome [73,74].

## 3. Microbial Risks of Livestock Excrement

The risks caused by microorganisms in livestock excreta are diverse, including direct risks caused by microorganisms themselves and indirect risks caused by secondary contamination generated by microbial processes. Therefore, limited exposure of humans and domestic animals and the surrounding environment to livestock excrement may be an effective strategy in avoiding the occurrence of associated microbial risks. An overall portrait of the microbial risks of livestock excrement is remarked below.

### 3.1. Pathogens Residing in Livestock Excrement

Many pathogens capable of infecting humans can be found in animal feces yet the feces from these animals pose a currently unquantified though likely substantial risk to human health. The insufficient separation of animal feces from human domestic environments can lead to the fecal–oral transmission of zoonotic pathogens through direct contact with animal feces or soil or fecal contamination of fomites, food, or water sources (Table 1).

There are some zoonotic viruses, including rotavirus, that were detected in livestock excrement samples [134]. Rotavirus infection is a common gastrointestinal illness caused by the rotavirus. It primarily causes diarrhea, vomiting, fever, and abdominal pain [135]. Rotavirus is highly contagious and spreads through the fecal–oral route, often through contaminated food, water, or surfaces [136]. And generally, the lack of good hygiene practices of livestock excrement accelerates the spread of rotavirus.

Being common opportunistic zoonotic pathogens, *Isospora*, *Cyclospora*, and *Microsporidia* were also reported to be found in livestock excrement, which causes infection and diarrhea in both humans and animals [137,138,139,140]. It was reported that sources of microsporidia species, such as *Enterocytozoon bieneusi*, that infect humans included major domestic animals such as horses [141], camels [142], goats [143], pigs [144], cows [144], rabbits [145], chickens [146], and donkeys [144] and the microsporidia species were mostly found in the faces of these animals; the vertical or transplacental transmission of microsporidiosis could occur in related animals and humans through the distribution of livestock excrement or related samples [147].

### 3.2. Transmission of Antibiotic-Resistant Genes

Antibiotic resistance is one of the serious threats to public health and food safety globally. Being a modern pollutant, antibiotic resistance genes (ARGs) are determined to be widely distributed in animal farms [148]. Antibiotic resistance bacteria and antibiotic-resistance genes enter the environment along with animal excrement, accelerating the spread of ARGs in the environment [149,150,151]. In the long run, antibiotic-resistant bacteria could be transmitted to humans through the food chain, water, or air, posing a great threat to public health.

Although it is very hard to obtain antibiotic use data in animal husbandry, these estimates are conservative as they were based on the baseline values of the global average annual consumption of antimicrobials per kilogram of animal produced. Every year 1.0 × 10^4^ to 2.0 × 10^5^ tons of antibiotics are used worldwide [4] of which their consumption in agriculture, especially the animal industries, occupies a significant fraction. And common antibiotic-resistant genes generally detected in livestock excrement were summarized in Table 2.

### 3.3. Toxic Chemicals Produced by Livestock Excrement Microbiome

Microorganisms in livestock excrement ferment substrates and produce numerous compounds. It has been reported that microorganisms in excrement ferment amino acids to produce toxic volatile substances such as indole, scatole, and a variety of alcohols and aldehydes [162,163,164]. Bacteria produce highly potent neurotoxins and resistant endospores [165,166] and these chemicals cause diseases in humans when exposed. 

*Candida albicans* were generally detected in livestock excrement; strains of this taxon could produce toxins such as *candidalysin* [167,168]. Candidalysin directly damages epithelial membranes, triggers a danger response signaling pathway, and activates epithelial immunity [169]. Candidalysin is also reported to promote alcohol-associated liver disease [170]. Pathogens like *Candida albicans* are prevalent in livestock excrements and during the manufacturing of the excrements these pathogens may enter the human and animal bodies as the food chain enters the human and animal bodies, causing related disease including infections (Table 3).

## 4. Strategies to Predict, Prevent the Microbial Risks of Livestock Excrement, and Beyond

### 4.1. Prediction of Livestock Excrement Microbial Risks

Effective prediction is a key strategy to prevent risks caused by microorganisms. Thanks to the rapid development of bioinformatics tools, such as next-generation sequencing (NGS) technology which includes 16S rRNA gene sequencing, shotgun metagenomic sequencing, and RNA sequencing, it has advanced our understanding of the microbiome by allowing for the discovery and characterization of microbes with the prediction of their function [189] which as a whole aided the development of predictive microbiology as well [190]. Notably, even though the microbial content of different environments is not static, there may exist certain patterns of each and these patterns could be explored by focusing on the risk-related node microbes of the microbiome of the system to further predict any potential microbial risks. Therefore, the relative microbial risk in animal excrement can be quickly estimated by these bioinformatics tools by focusing on well-documented pathogens and those taxa which contain any genes that code proteins with determined pathogenic potential, ideally by using metagenomic sequencing of all microorganisms (viruses, prokaryotes, and eukaryotes), amplicon sequencing to determine bacteria, and ITS sequencing to determine fungi [191].

Moreover, target-specific primers can be designed for any well-documented pathogenic microorganisms and the absolute abundances of target pathogenic microorganisms (including viruses, bacteria, fungi, and parasites) can be quantified by the quantitative polymerase chain reaction (qPCR). Reports showed that by using qPCR, *Brucella melitensis* and *Brucella abortus* in different samples, including raw milk and cheese, could be determined accurately so as to predict the potential health adversity of the corresponding samples [192,193,194]. And zoonotic parasites including *Toxoplasma gondii* were also determined using qPCR so as to prevent any potential risks that might be caused [195].

In addition, certain novel tools could be applied to predict the microbial risks that could be caused by livestock excrements. Artificial intelligence tools including machine learning and deep learning were generally considered useful aids to predict microbial pandemic cases and the spread patterns of the microbes [196]. Reports find that machine learning algorithms can be used to predict the host of the influenza virus and the identification of influenza virus host range and zoonotic transmissible sequences [197,198]. Therefore, these modern tools could also be applied to predict the threats that might be caused by the microbiome of livestock excrement and the probable fate of the related risky microbes.

### 4.2. Prevention and Management of Livestock Excrement Microbial Risks

Pathogens in livestock excrement cause diseases if they are not properly managed. Therefore, preventing and managing microbial risks associated with livestock excrement are essential for maintaining food safety and protecting public health.

To prevent microbial risks, the livestock and poultry environments should be frequently and sufficiently sanitized and livestock should receive necessary vaccinations to avoid the transmission of risky microorganisms [199]. When any unfortunate microbial risk occurs, blocking the route of transmission is the most effective strategy for control [200]. An important part of this is protecting humans during farming activities. In addition, free-range breeding of livestock can easily cause the rapid spread of risky microorganisms, thereby increasing the risk of microorganisms. Therefore, captivity should be promoted vigorously when grazing.

Secondly, appropriate disposal measures should be established for risks caused by different microbial taxa. In the case of viral microbial risks, causative viruses should be eradicated by sanitizing with agents in livestock excreta and related environments. Risks posed by pathogenic bacteria and fungal microorganisms can be eradicated through the use of bactericides and fungicides which should be conducted under the supervision of qualified veterinarians after a solid diagnosis regarding the occurring issue to determine the severity of the event and design an efficient strategy or protocol. A series of *N*-aryl-pyridine-4-one derivatives were reported to show fungicidal/bactericidal activities and their fungicidal activities action against *Colletotrichum* were investigated [201]. The application of these anti-pathogenic agents after careful selection and usage could aid the prevention of the microbial threats that could be caused by livestock excrement microbes. Parasitic pathogens can be sterilized through treatment with pesticides such as dihydroartemisinin and piperaquine [202]; bactericides and fungicides such as chlorhexidine and hydrogen peroxide can also be used to kill pathogenic bacteria and fungi [203,204,205]. 

However, it should be kept in mind that the application of these anti-pathogenic agents, pesticides and insecticides mentioned above should be considered carefully to prevent potential secondary pollution. While these agents can be effective in combating various pathogens and preventing infections, there are important factors to take into account. The potential side effects and risks associated with the use of these agents should be carefully evaluated. Some agents, including dichlorodiphenyltrichloroethane (DDT) [206], organophosphate insecticides, carbamates, and pyrethroid insecticides [207] have adverse effects on human health or the environment. Ultimately, a comprehensive risk-benefit analysis should guide the decision-making process when considering the application of these agents. Hence, certain well-documented safer agents, including plant-extracted anti-insect agents (such as neem oil and canola oil) [208,209] and microbial pesticides (such as the mosquitocidal agents from *Bacillus sphaericus*) [210] should be considered in livestock excrement treatment.

Moreover, it is essential to sterilize the closely related components, such as water, by chlorination for controlling harmful pathogens [211]. And the farming environments where pathogenic bacteria occur could be sterilized by using high-concentration acid and alkali treatments and high temperatures (such as burning). In addition, to prevent the spread of pathogenic bacteria, safer and more effective disposal measures still need to be developed. Moreover, insecticides should be used to prevent mosquito-borne or mosquito-transmitted diseases [212,213] so as to significantly prevent the spread of pathogens and parasites.

## 5. Limitations of the Current Microbial Research of Livestock Excrement

Even though current microbial research on livestock excrement has already shown a crucial role in understanding the ecological impact and their potential risks, microbial research in livestock excrement faces several limitations, including the complexity and diversity of microbial communities, spatial and temporal heterogeneity, the lack of standardization, and limited longitudinal studies.

One significant limitation is the sheer complexity and diversity of microbial communities present in livestock excrement. The excrement microbiome is composed of a vast array of microorganisms; studying these diverse communities and understanding their interactions and functions is a challenging task due to technological and methodological constraints. Generally, culture-dependent techniques may only capture a fraction of the microbial diversity, leading to an incomplete understanding of the overall ecosystem [214]. Furthermore, sampling and spatial heterogeneity pose additional challenges in microbial research [215]. Livestock excrement is inherently variable, with microbial communities varying across animal species, diets, management practices, and environmental conditions. Obtaining representative samples that accurately reflect the entire excrement microbial population can be difficult, especially when considering the large-scale production systems commonly found in the livestock industry. Additionally, temporal dynamics must be accounted for as microbial populations can change over time, impacting our ability to form a comprehensive understanding.

Another limitation is the lack of standardization in experimental design and methodologies across studies regarding the microbiome of livestock excrement. Inconsistent sampling and analysis protocols lead to the comparability and reproducibility of the corresponding results [216,217]. This variability makes it challenging to draw meaningful conclusions and make accurate comparisons between different research studies. Hence, standardization efforts and collaborations among researchers are needed to establish the best practices and to enhance the reliability and validity of microbial research in livestock excrement. Moreover, there is a need for more longitudinal studies which can provide valuable insights into the effects of management practices, interventions, and seasonal variations to better understand the dynamic nature of microbial communities in livestock excrement [218]. Many studies focus on short-term assessments, providing only a snapshot of the microbial composition at a particular moment.

In conclusion, addressing these limitations through improved methodologies, standardization efforts, and collaborative research endeavors can enhance our understanding of the microbial ecology of livestock excrement and help in developing effective management strategies that minimize the environmental impact and mitigate associated risks.

## 6. Conclusions

Livestock excrement is a major pollutant yield from farming and is constantly imported and transferred into every related environment. The livestock excrement microbiota comprises a variety of microorganisms of viruses, prokaryotes, and eukaryotes and these microorganisms are activated and transferred synchronically during the processes of livestock excrement production, transformation, and utilization. The livestock excrement microbiome is extensively affecting and affected by the microbiome of humans and the relevant environments. The zoonotic microbes, extremely zoonotic pathogens, and antibiotic-resistant bacteria are posing threats to human health and environmental safety. 

In this review, we highlighted the main features of the microbiome of livestock excrement and elucidated the composition and structure of the repertoire of microbes, how these microbes transfer from different spots, how they affect the microbiomes of the habitants in any related environments, and what risks they may cause in humans, animals and the environment as a whole. Overall, the environmental problems caused by the microbiome of the livestock excrement and the potential risks it may cause were summarized from the microbial perspective. In addition, the prevalence of antibiotic resistance and virulence genes in major livestock excrement microbes were summarized so as to provide a reference for further studies regarding potential microbial risks of livestock excrement microbes.

## Figures and Tables

**Table 1:** Common zoonotic pathogens detected in livestock excrement.

Pathogen	Host of Livestock Excrement	Disease Caused	References
Rotavirus	Sheep, goat, cattle, pig	Diarrhea, vomiting, fever, abdominal pain	[75,76,77,78]
*Echinococcus granulosus*	Camel, horse, sheep, pig	Hydatidosis	[79,80]
*Pasteurella multocida*	Sheep, goat, deer, pig, cattle, chicken	Fowl cholera	[81,82,83,84]
*Brucella melitensis*	Goat, sheep, cattle, camel	Brucellosis	[85]
*Brucella abortus*	Camel, cattle	Brucellosis	[86,87]
*Bordetella bronchiseptica*	Sheep, pig, goat	Whooping cough	[88,89]
*Malassezia pachydermatis*	Horses, camel, cattle, poultry, sheep, goat, rabbit	Dermosis	[90]
*Leptospira* sp.	Sheep, cattle, goat, horse,	Reproductive failures and infertility	[91,92,93]
*Campylobacter* sp.	Sheep, chicken	Infection, abortion	[94]
*Mycobacterium tuberculosis*	Sheep, cattle	Tuberculosis	[95,96]
*Staphylococcus pseudintermedius*	Sheep, goat	Dermatological disease, cow mastitis	[97,98]
*Clostridium difficile*	Cattle, sheep, horse, and goat, poultry	*Clostridium difficile* infection	[99]
*Enterocytozoon bieneusi*	Sheep, goat, cattle, camel, pig, yak, chicken, horse, rabbit	Diarrhea	[100,101,102,103,104,105,106,107,108]
*Plasmodium falciparum*	Cattle, goat, pig, poultry	Malaria	[109]
*Giardia lamblia*	Sheep, goat, cattle	Giardiasis	[39,40]
*Giardia duodenalis*	Cattle, deer, pig, goat, horse, sheep, chicken, yak	Giardiasis	[110,111,112,113,114,115,116]
*Salmonella* spp.	Sheep, cattle, chicken, horse	Diarrhea, loss of appetite, fever, depressed mentation, mortality	[117,118,119,120]
*Yersinia enterocolitica*	Sheep, cattle, pig	Yersiniosis; Enteritis	[39,121,122]
*Listeria monocytogenes*	Sheep, cattle, horse, chicken	Listeriosis	[123,124,125,126]
*Legionella pneumophila*	Pig	Legionnaires’ disease	[127]
*Staphylococcus saprophyticus*	Cattle	Urinary tract infection	[128]
*Haemophilus ducreyi*	Pig	Chancroid	[129]
*Toxoplasma gondii*	Sheep, goat, pig, chicken	Toxoplasmosis	[130]
*Trichinella*	Cattle, sheep, horse	Trichinellosis	[131,132,133]

**Table 2:** Antibiotic-resistant genes are generally detected in livestock excrement.

Gene	Resistant Antibiotic	Related Excrement Samples	References
*tet*	Tetracycline resistance	Swine, cattle, poultry manure	[152,153]
*sul*	Sulfonamide resistance	Swine manure	[154]
*erm*	Erythromycin resistance	Swine wastewater	[155]
*fca*	Fluoroquinolone, quinolone, florfenicol, chloramphenicol, and amphenicol (FCA) resistance	Cattle manure, swine manure	[156,157]
*bla*	*β*-lactamase resistance	Poultry manure	[158]
*mdr*	Aminoglycosides resistance	Swine manure	[159]
*van*	Vancomycin resistance	Poultry manure, swine manure, cattle manure	[158,160,161]

**Table 3:** Toxic chemicals produced by livestock excrement microbiome.

Chemicals	Health Risk	Related Samples	References
Indole	Colorectal cancer, bipolar disorder	Swine waste	[171,172]
*p*-Cresol	Kidney and liver damage	Swine waste, sheep manure	[173,174]
Skatole	Respiratory distress	Swine waste, goat, sheep and cattle manure, poultry manure	[175,176,177]
Phenols	Skin irritation, respiratory disorder	Swine waste, poultry manure	[172,178]
Hydrogen sulfide	Respiratory disorder	Swine manure, poultry manure	[179,180]
Ammonia	Skin and eye irritation, respiratory disorder	Swine manure, cattle manure, poultry manure	[181,182,183,184]
Heavy metals (Cu, Pb, Hg, Cd, As)	Liver and kidney damage	Swine manure, cattle manure	[185,186,187,188]

## Data Availability

There is no data created in this manuscript, and for any cited datasets, we have already provided the corresponding references, for Figures and Tables.
